# Divergent G-protein selectivity across melanopsins from mice and humans

**DOI:** 10.1242/jcs.258474

**Published:** 2022-03-21

**Authors:** Richard J. McDowell, Jessica Rodgers, Nina Milosavljevic, Robert J. Lucas

**Affiliations:** Centre for Biological Timing, Division of Neuroscience and Experimental Psychology, Faculty of Biology Medicine and Health, University of Manchester, Manchester M13 9PT, UK

**Keywords:** G-protein-coupled receptor, Melanopsin, Optogenetics, Photoreceptor

## Abstract

Melanopsin is an opsin photopigment and light-activated G-protein-coupled receptor; it is expressed in photoreceptive retinal ganglion cells (mRGCs) and can be employed as an optogenetic tool. Mammalian melanopsins can signal via G_q/11_ and G_i/o/t_ heterotrimeric G proteins, but aspects of the mRGC light response appear incompatible with either mode of signalling. We use live-cell reporter assays in HEK293T cells to show that melanopsins from mice and humans can also signal via G_s_. We subsequently show that this mode of signalling is substantially divergent between species. The two established structural isoforms of mouse melanopsin (which differ in the length of their C-terminal tail) both signalled strongly through all three G-protein classes (G_q/11_, G_i/o_ and G_s_), whereas human melanopsin showed weaker signalling through G_s_. Our data identify G_s_ as a new mode of signalling for mammalian melanopsins and reveal diversity in G-protein selectivity across mammalian melanopsins.

## Abstract (precis)

**Summary:** The photopigment melanopsin (OPN4), which provides inner retinal photoreception in mammals, shows light-dependent activation of G_s_ G protein that is more pronounced for mouse than human photopigment.

## INTRODUCTION

A fraction of mammalian retinal ganglion cells (mRGCs) are directly light responsive due to expression of the photopigment melanopsin. These mRGCs contribute to a variety of light responses, from circadian photoentrainment to perceptual vision (Aranda and Schmidt, 2020; Do, 2019). Melanopsin is a light-activated G-protein-coupled receptor (GPCR) and a member of the animal opsin family. Melanopsin couples to native G-protein signalling cascades to drive light responses under heterologous expression in non-photosensitive cells (Melyan et al., 2005; Panda et al., 2005; Qiu et al., 2005), leading to its increasing use in the development and application of optogenetic tools (Beiert et al., 2014; De Silva et al., 2017; Lin et al., 2008; Mederos et al., 2019; van Wyk et al., 2015). Mammalian melanopsins show high sequence conservation. One exception is the C-terminal region, where differences exist both between species and within mice due to the existence of splice variants with different C-terminal extensions (Lang et al., 2021; Pires et al., 2009). Further sequence divergence between species has been identified in intracellular loop 3 (IL3), which along with the C terminus, is thought to play an important role in G-protein interaction (Pires et al., 2007; Valdez-Lopez et al., 2020b).

The G-protein signalling cascades engaged by melanopsin are central to its native physiological functions and optogenetic applications. The G_q/11_–phospholipase C cascade is an important component of the intrinsic light response of mRGCs (Graham et al., 2008; Hartwick et al., 2007; Xue et al., 2011), and mammalian melanopsin is often referred to as a G_q/11_-coupled receptor. However, there is evidence that mammalian melanopsins also signal via other G proteins and, at least *in vitro* or under heterologous expression, show light-dependent coupling to G proteins of the Gα_i/o/t_ family (Bailes and Lucas, 2013; Newman et al., 2003; Spoida et al., 2016).

Recent studies of mRGC physiology have led to renewed interest in the G-protein signalling partners of melanopsin. Despite evidence that melanopsin couples to Gα_q/11_, genetic disruption of members of the G_q/11_ family (Gα_q_, Gα_11_, Gα_14_) does not eliminate the mRGC intrinsic light response (Chew et al., 2014). The established Gα_i/o/t_ activity of melanopsin is unlikely to drive the ‘non-G_q/11_ light response’, as G_i/o_ pathways generally inhibit neurons. Jiang and colleagues (Jiang et al., 2018) have presented evidence that light drives an increase in cyclic nucleotides [especially cyclic AMP (cAMP)] in some mouse mRGC subtypes, raising the possibility that melanopsin also activates Gα_s_. Mouse mRGCs express Gα_s_, and it has recently been proposed that the ability to couple to both G_i/o_ and G_s_ may be a common feature of G_q_-coupled GPCRs (Okashah et al., 2019; Peirson et al., 2007). We set out here, therefore, to use a heterologous expression system to determine whether mammalian melanopsins do indeed show light-dependent interaction with Gα_s_. Having addressed this potential mode of signalling, we then wished to ask how well G-protein selectivity was conserved across structurally divergent mammalian melanopsins.

## RESULTS AND DISCUSSION

### Human melanopsin shows light-dependent coupling to both Gα_i/o_ and Gα_s_

**Fig. 1.**
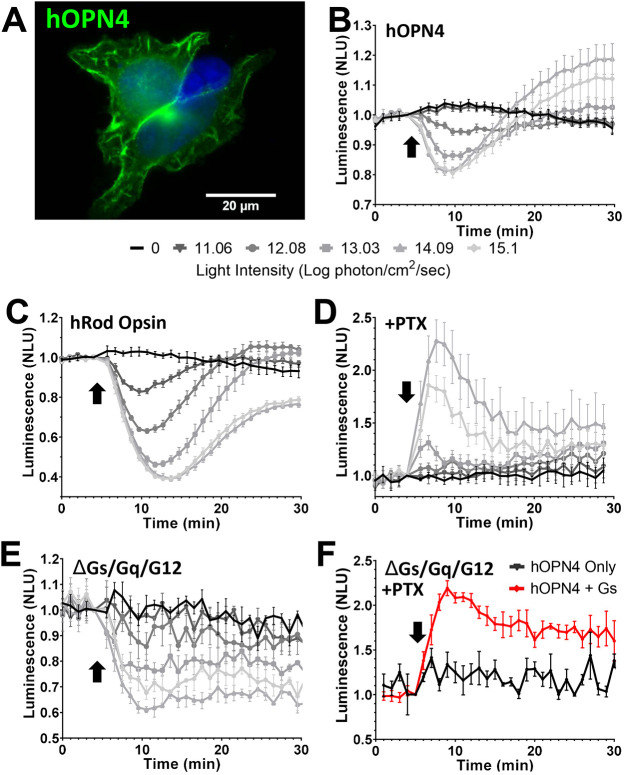
**Human melanopsin shows light-dependent coupling of both Gα_i/o_ and Gα_s_.** (A) Immunocytochemistry photomicrograph showing human OPN4 (green) in HEK293T cells (DAPI-stained nuclei, blue). (B–F) Changes in bioluminescence in response to a 1 s 470 nm light flash (arrow), normalised to 1 at the time of the light pulse, from HEK293 cells expressing the cAMP reporter GloSensor and either hOPN4 (B,D–F) or hRod Opsin (C). (B–E) Responses across a range of flash intensities for hOPN4 (B) and hRod Opsin (C) in HEK293T cells, hOPN4 in HEK293T cells treated with pertussis toxin (PTX) to eliminate G_i/o_ signalling (D) and hOPN4 in HEK293 ΔGs/Gq/G12 cells (E). Key for light intensity in B–E is shown below B (B and C, *n*=4; D and E, *n*=3). (F) hOPN4-expressing HEK293 ΔGs/Gq/G12 cells treated with PTX with (hOPN4+Gs) or without (hOPN4 only) heterologous Gα_s_ exposed to a 1s 14.09 log photon/cm^2^/sec light flash (hOPN4 only, *n*=3; hOPN4+Gs, *n*=5). Data expressed as mean±s.e.m. Cells pretreated with 2 µM forskolin to elevate the starting level of cAMP for traces in B,C,E. NLU, normalised luminescence units. *n* values denote biological replicates from independent transfections.

Genetically encoded bioluminescent second-messenger reporters represent a powerful approach to reveal light-dependent opsin signalling (Bailes and Lucas, 2013; Koyanagi et al., 2013). These can be applied in live cells, overcoming technological challenges of purifying functional opsin for traditional biochemical analyses. Here, we first adopted this approach using GloSensor, a luminescent cAMP reporter, because G_s_ signalling results in an increase in this second messenger. HEK293T cells expressing GloSensor and human melanopsin (hOPN4; Fig. 1A) responded to a 1 s 470 nm light flash (at intensities of ≥12.08 log photon/cm^2^/sec) with a reduction in luminescence following pre-treatment with forskolin (Fig. 1B), as previously reported (Bailes and Lucas, 2013) and consistent with the known ability of melanopsin to suppress cAMP via G_i/o_ signalling. However, at higher flash intensities (≥13.03 log photon/cm^2^/sec), this initial suppression was followed by a rebound overshoot ∼13 min following the light stimulus (Fig. 1B).

We confirmed that the rebound overshoot in GloSensor luminescence in hOPN4-expressing cells was not a product of Gα_i/o_ signalling in two ways. Firstly, we showed that it was not replicated in cells expressing human rod opsin (hRod Opsin, also known as RHO), which shows effective Gα_i/o_-coupling in these cells (Fig. 1C). Secondly, application of the Gα_i/o/t_ inhibitor, pertussis toxin (PTX), abolished the initial cAMP decrease, but not the subsequent increase in cAMP (Fig. 1D).

There are at least two plausible routes via which melanopsin could drive increases in cAMP: direct G_s_ pathway activation, or crosstalk from the G_q_ or G_12_ signalling pathways (Gupte et al., 2017; Jiang et al., 2008; Patel et al., 2001). To determine whether the cAMP increase originated with Gα_s_ we used a knockout cell line (Grundmann et al., 2018). hOPN4-expressing HEK293 cells engineered to lack Gα_s_, Gα_q_ and Gα_12_ (hereafter referred to as ΔGs/Gq/G12 cells) retained the initial light-dependent reduction in GloSensor luminescence but lacked the subsequent recovery or overshoot (Fig. 1E). PTX blocked all GloSensor responses in this cell line, consistent with the view that the remaining light response originated with Gα_i/o_ (Fig. 1F). A light-dependent increase in cAMP could be recovered by introducing heterologous Gα_s_, confirming that Gα_s_ is both necessary and sufficient for the observed hOPN4-dependent increases in cAMP (Fig. 1F).

**Fig. 2.**
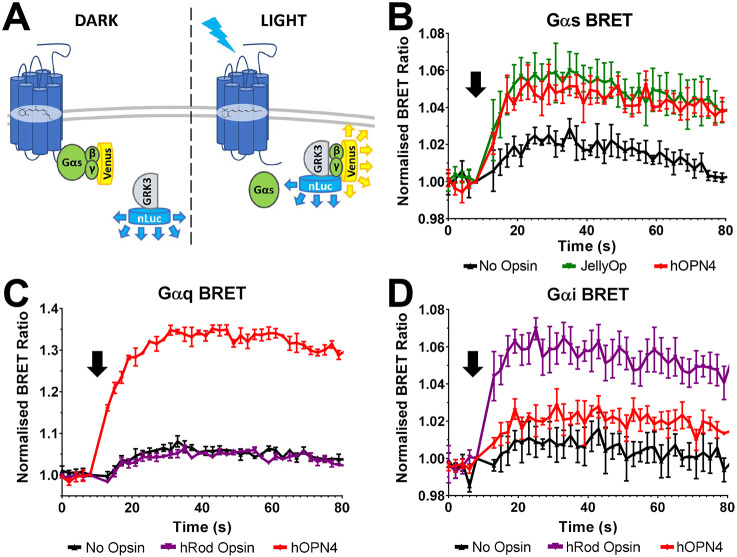
**hOPN4 coupling to Gα components as revealed by Gα BRET assay.** (A) Schematic of BRET assay components and interactions. Exogenous hOPN4 is co-expressed with BRET components: Gα, Gβγ–Venus and GRK3–nLuc. In the dark (left), hOPN4 is inactive and Gβγ–Venus is sequestered in a heterotrimer with Gα. Upon light activation (right), hOPN4 drives dissociation of the heterotrimer to release free Gβγ–Venus that can be bound by GRK3–nLuc. Close interaction between GRK3–nLuc and Gβγ–Venus results in BRET between nLuc (donor) and Venus (acceptor). (B–D) Change in BRET ratio (normalised to 1 at time of light flash) in HEK293 ΔGs/Gq/G12 cells expressing hOPN4 (red), JellyOp (green) or hRod Opsin (purple), and BRET assay components including Gα_s_ (B), Gα_q_ (C) or Gα_i(ser)_ (D) in response to a 1 s 470 nm light flash (16.1 log photon/cm^2^/sec, at arrow). Data expressed as mean±s.e.m. *n*=3 biological replicates from independent transfections.

As a final demonstration of Gα_s_ coupling to hOPN4, we employed a bioluminescence resonance energy transfer (BRET)-based interaction assay (Fig. 2A) (Masuho et al., 2015). In brief, PTX-treated HEK293 ΔGs/Gq/G12 cells were co-transfected with expression vectors for: hOPN4, Gα_s_, Gβ and Gγ tagged with a split-Venus fluorophore (Gβγ–Venus), as well as a modified C-terminal fragment of G-protein-coupled receptor kinase 3 (GRK3ct) covalently linked to the bioluminescent luciferase Nanoluc (GRK3–nLuc). In these cells, heterologously expressed Gα_s_ and Gβγ–Venus are expected to form a heterotrimer sequestering the Venus fluorophore away from the Nanoluc tagged to GRK3. If hOPN4 were able to activate Gα_s_, light stimulus should lead to dissociation of the G-protein heterotrimer, releasing Gβγ–Venus to bind to GRK3–nLuc and convey an increase in the BRET ratio.

We found that light induced a small change in BRET even in cells lacking opsin (possibly reflecting partial bleaching of BRET assay components). Nevertheless, light stimuli induced a much larger increase in BRET in hOPN4-expressing cells, confirming that hOPN4 shows light-dependent activation of Gα_s_ (Fig. 2B). This event was larger than any change observed in cells lacking hOPN4 and was equivalent to that in cells expressing the Gα_s_-coupled opsin, JellyOp (Kruskal–Wallis test *H*=58.3, *P*<0.0001; Dunn's multiple comparison post hoc test, JellyOp *P*<0.0001, hOPN4 *P*<0.0001). We further applied this assay to confirm that hOPN4 can couple with Gα_q/11_ and Gα_i/o/t_ G-proteins by replacing the Gα_s_ expression vector with one driving expression of either Gα_q_ or a PTX-insensitive version of Gα_i_ (Fig. 2C,D). In both cases, light pulses drove increases in BRET ratio for hOPN4-expressing cells (Kruskal–Wallis test *H*=57.40, *P*<0.0001 for Gα_i_; *H*=77.70, *P*<0.0001 for Gα_q_; Dunn's multiple comparison post hoc test, *P*<0.0001 for Gα_q_ hOPN4, *P*<0.0001 for Gα_i_ hOPN4). hRod Opsin behaved as expected and drove significant increases in BRET ratio for Gα_i_ (Gα_i_ hRod Opsin *P*<0.0001) but not Gα_q_ (Gα_q_ hRod Opsin *P*=0.34) demonstrating the ability of the BRET assay to convey G-protein specificity. In summary, these experiments confirm that human melanopsin is capable of light-dependent activation of Gα_s_, in addition to its documented ability to activate Gα_q/11_ and Gα_i/o/t_ G proteins.

### Gα_s_ signalling in mouse melanopsin

**Fig. 3.**
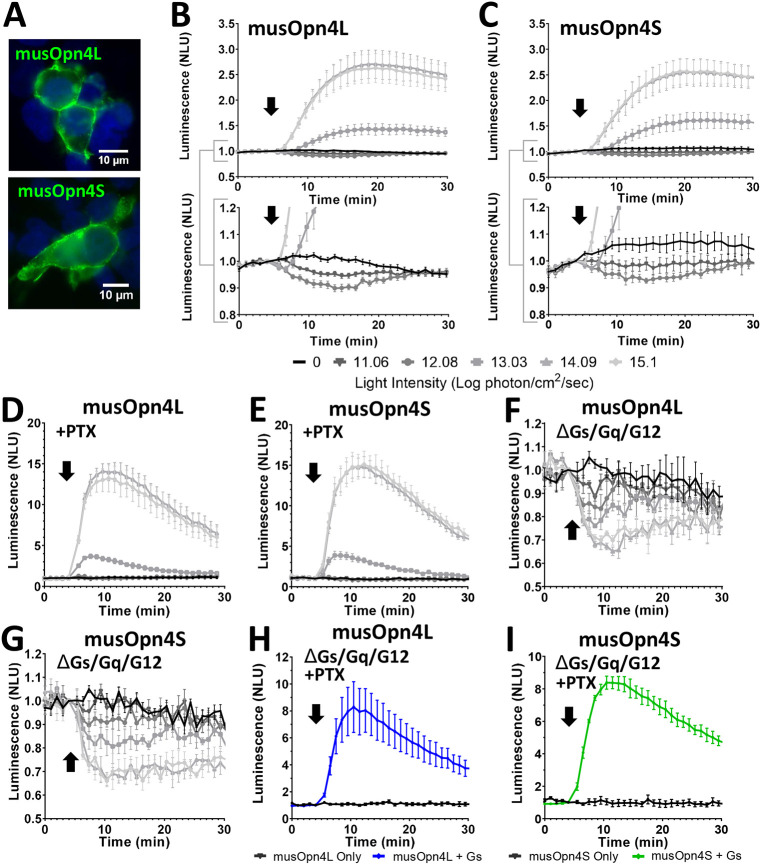
**Gα_s_ signalling in splice variants of mouse melanopsin.** (A) Immunocytochemistry photomicrograph showing musOpn4L (green, top) and musOpn4S (green, bottom) in HEK293T cells (DAPI-stained nuclei, blue). (B–I) Changes in bioluminescence in response to a 1s flash of light (arrow), normalised to 1 at the time of stimulus, from HEK293 cells expressing the cAMP reporter GloSensor and either musOpn4L (B,D,F,H) or musOpn4S (C,E,G,I). (B,C) Responses across a range of flash intensities (key below the graphs) for musOpn4L (B, top) and musOpn4S (C, top). *n*=4. Brackets indicate regions shown beneath with constrained *y* axes. (D,E) HEK293T cells treated with PTX to eliminate G_i/o_ signalling or (F,G) HEK293 ΔGs/Gq/G12 cells exposed to a 470 nm flash at a range of light intensities (key below B and C). D, *n*=4; E, *n*=5; F, *n*=3; G, *n*=4. (H,I) HEK293 ΔGs/Gq/G12 cells treated with PTX with (blue in H, green in I) or without (black) transfection of a Gα_s_ expression vector, exposed to a 1 s 470 nm 14.09 log photon/cm^2^/sec light flash (H, *n*=4; I, *n*=4). Data expressed as mean±s.e.m. Cells pretreated with 2 µM forskolin to elevate starting level of cAMP for traces in B,C,F,G. NLU, normalised luminescence units. *n* values denote biological replicates from independent transfections.

Differences in mRNA splicing produce two melanopsin isoforms in mice that are identical over the bulk of the protein (455 amino acids) but divergent in the C-terminal region, which comprises 12 amino acids in the shorter isoform (musOpn4S) and 67 amino acids in the longer (musOpn4L) (Fig. S1) (Pires et al., 2009). While there is evidence that musOpn4S and musOpn4L support different visual responses (Jagannath et al., 2015), to date no differences have been identified in the signalling profiles of the isoforms. Both isoforms of mouse melanopsin expressed well in HEK293T cells (Fig. 3A; Fig. S2). Interestingly, neither isoform replicated the marked biphasic GloSensor flash response produced by hOPN4 in these cells (Fig. 3B,C). In cells expressing either isoform, bright light pulses (≥10^13^ photon/cm^2^/sec) produced a simple increase in GloSensor luminescence, whereas a small reduction in luminescence was revealed only at lower light intensities (Fig. 3B,C). Increases in GloSensor luminescence survived application of PTX in both musOpn4L- and musOpn4S-expressing cells (Fig. 3D,E), consistent with the conclusion that both opsins displayed G_s_ activity. In further agreement with this interpretation, increases in luminescence were lost in ΔGs/Gq/G12 cells (Fig. 3F,G) and restored by reintroducing Gα_s_ (Fig. 3H,I).

### Differences in G-protein selectivity between human and mouse melanopsins

**Fig. 4.**
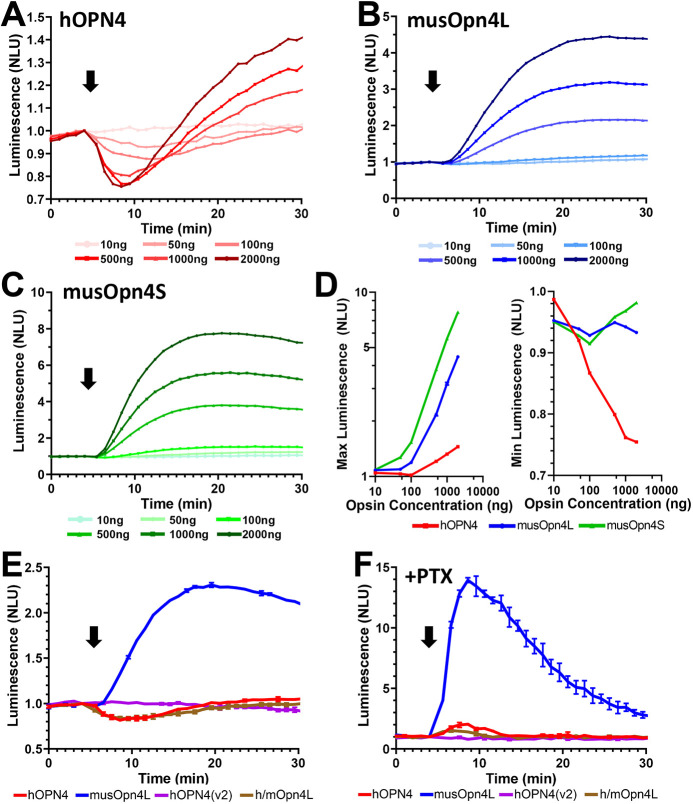
**Divergent G-protein preference across melanopsins.** (A–C) Time courses of light-induced cAMP changes revealed by GloSensor luminescence in HEK293T cells transfected with 10 ng to 2000 ng of plasmid expression vector for (A) hOPN4, (B) musOpn4L or (C) musOpn4S when stimulated with a 1 s 470 nm 14.09 log photon/cm^2^/sec light flash (arrow). Mean of four technical replicates. (D) Dose response curves derived from maximum (left) or minimum (right) values obtained from A–C. hOPN4 (red), musOpn4L (blue) and musOpn4S (green). Mean of four technical replicates. (E,F) Changes in bioluminescence in response to a 1 s flash of 14.09 log photon/cm^2^/sec 470 nm light (arrow), normalised to 1 at time of light pulse, from HEK293 cells expressing the cAMP reporter GloSensor and either hOPN4 (red), musOpn4L (blue), h/mOpn4L (brown) and hOPN4(v2) (purple) in HEK293T cells in the absence (E) or presence (F) of pertussis toxin. *n*=2 biological replicates from independent transfections. Data expressed as mean±s.e.m. Cells pretreated with 2 µM forskolin to elevate starting level of cAMP for traces in A,B,C,D and E. NLU, normalised luminescence units.

A substantial difference in G_s_ response between the human and mouse melanopsins is apparent from a cursory examination of their GloSensor response (Figs 1B and 3B,C). Despite the high levels of Gα_s_ reported in HEK293T cells, G_i/o_ signalling was more reliably observed in hOPN4-expressing cells, with reductions in cAMP apparent at earlier timepoints and lower light intensities. Conversely the mouse melanopsins drove large increases in cAMP, indicating strong G_s_ activity, which overwhelmed any G_i/o_ responses except at very low irradiances. One possibility for the difference in response preference between melanopsins could be that human melanopsin expresses more poorly in our experimental system. Immunocytochemistry indicated that all opsins could be effectively expressed in HEK293 cells (Fig. S2). However, it remains possible that the fraction of properly folded and fully functional opsin differs between melanopsins. We therefore set out to determine the extent to which our conclusions were robust to variations in melanopsin content, by varying the quantity of expression plasmid used for transfection. We found that the magnitude of the light response elicited by the three melanopsins was strongly determined by plasmid concentration, consistent with the prediction that there was a corresponding change in the amount of functional opsin expressed (Fig. 4A–D). However, the fundamental bias of hOPN4 towards G_i/o_ signalling was retained across all plasmid concentrations (Fig. 4A,D). Thus, the initial decrease in cAMP was the most robust component of the hOPN4 light response, appearing at lower plasmid concentrations and preceding the subsequent cAMP increases at higher plasmid concentrations (Fig. 4A,D). Conversely, increases in cAMP were the dominant component of musOpn4L and musOpn4S light responses across all plasmid concentrations (Fig. 4B–D). Thus, when human and mouse melanopsins have access to both G_i/o_ and G_s_ signalling (in HEK293T cells), the relative magnitude of their interaction across these two G-protein classes is species dependent.

The hOPN4 employed in this study represents the only melanopsin isoform currently identified in human retina. Nevertheless, we wished to determine whether other putative isoforms of hOPN4 had more substantial G_s_ coupling. Analysis of the human genome indicates the possibility of a melanopsin splice variant with an extended intracellular loop 1 (IL1) region [termed here hOPN4(v2); Fig. S1]. We therefore synthesised this putative isoform and applied it to our assay system. hOPN4(v2) was unable to drive a cAMP light response in HEK293T cells (Fig. 4E,F), indicating either that it cannot drive light-dependent activation of either G_i/o_ or G_s_ cascades, or that it does not function in our assay system. Finally, we addressed the possibility that a human version of the musOpn4L isoform may exist (i.e. with an extended C terminus) by constructing a chimera (h/mOpn4L) in which the terminal 66 amino acids of musOpn4L (from residue 455Q) were appended to hOPN4 (from residue 457Q) (Fig. S1). This h/mOPN4L successfully elicited light-dependent changes in cAMP in HEK293T cells (Fig. 4E,F). The pattern of G_i/o_ and G_s_ signalling produced by h/mOPN4L was broadly similar to that produced by hOPN4 and was different to that of musOpn4L. Thus, the light response was dominated by a reduction in cAMP indicative of dominant G_i/o_ activity (Fig. 4E). A modest increase in cAMP was revealed following PTX administration, which is consistent with some G_s_ activity, but the magnitude was qualitatively different to that achieved with musOpn4L (Fig. 4F). Taken together these data provide confidence that the relative bias against G_s_ signalling of hOPN4 would be retained for any putative hOPN4L isoform.

The data presented here represent the first confirmation that mammalian melanopsins are capable of signalling through the G_s_ signalling pathway. Circumstantial evidence supports the notion that mouse melanopsin engages G_s_ pathways in mRGCs. There are six anatomically distinguishable classes of mRGC in mice (M1–M6) (Do, 2019). M1, M2 and M4 cells have a G_q/11_ light response (Jiang et al., 2018; Sonoda et al., 2018), as predicted given the G_q_ signalling ability of musOpn4L and musOpn4S described here (Fig. 4). However, alternative HCN channel signalling driven by increases in cAMP has been ascribed to M2 (and M4) but not M1 cells (Jiang et al., 2018). Furthermore, M2 and M4 cells express lower levels of Ras guanyl nucleotide-releasing protein 1 (also known as RASGRF1), a guanine-nucleotide-exchange factor (GEF) that binds to second messengers of G_q_ signalling, diacylglycerol and Ca^2+^ (Berg et al., 2019). Taken together, these observations suggest that melanopsin-mediated G_s_-driven cAMP increases may be responsible for the signalling observed in M2 and M4 mRGCs.

The similar G-protein response preference observed for the two murine melanopsin isoforms in our test systems suggests that previously observed differences in the visual consequences of disrupting musOpn4S versus musOpn4L expression (Jagannath et al., 2015) do not reflect differences in fundamental signalling activity of these melanopsins. Rather, divergence in the visual responses elicited by these pigments could arise from differences in the degree to which mRGC subtypes rely on each isoform and/or some other aspect of their biology (Jagannath et al., 2015; Masuho et al., 2015; Pires et al., 2009; Valdez-Lopez et al., 2020a).

The discovery of divergent G-protein selectivity across mammalian melanopsins has important implications for the increasing use of melanopsin as an optogenetic tool. Despite the tendency to regard melanopsin as a tool for modulating G_q_ signalling, all three melanopsins showed activation of G_i/o_ and G_s_ pathways. This was particularly the case for murine melanopsin, whereas hOPN4 exhibited weaker G_s_ signalling. In future, the natural variation in G-protein selectivity across melanopsins from mammals (and other vertebrates) could provide fertile ground for engineering variants with selective G-protein activity.

## MATERIALS AND METHODS

### Construction of expression vectors

Open reading frames for opsins (human OPN4, NM_033282.4; mouse OPN4S, NM_001128599.1; mouse OPN4L, NM_013887.2; human rhodopsin, NM_000539.3; JellyOp, AB435549; hOPN4L, NM_001030015.3) tagged with 1D4 C-terminal epitope, luminescent reporters and BRET assay components were introduced into the multiple cloning site of the pcDNA3 vector (Invitrogen) downstream of the CMV promoter. Chimeric hOPN4 with musOpn4L C-terminal tail (h/mOpn4L) was generated by replacing the C-terminal section of hOPN4 from 457Q with the C-terminal tail of musOpn4L from 455V.

### Luminescent second-messenger assays

Freshly thawed and validated HEK293T (American Type Culture Collection) or HEK293 ΔGs/Gq/G12 KO cells (kindly provided by Prof. Asuka Inoue, Tohoku University, Japan) were cultured in Dulbecco's modified Eagles medium (4.5 g l^−1^ D-glucose, sodium pyruvate and L-glutamine with 10% foetal calf serum; DMEM) were transiently transfected with plasmid expression vectors for the relevant opsin (500 ng, unless otherwise stated) and genetically encoded indicator bioluminescent cAMP reporter (GloSensor, 500 ng; Promega) using Lipofectamine 2000 (Thermo Fisher Scientific) and incubated overnight with 10 µM 9-*cis*-retinal (Sigma-Aldrich) and, where relevant, 100 ng/ml pertussis toxin (PTX; Sigma-Aldrich) (as described previously in Bailes and Lucas, 2013). They were then incubated with 2 mM beetle luciferin (Sigma-Aldrich) substrate at room temperature for 30 min before being transferred to the plate reader (Optima FLUOStar, BMG), and, where relevant, application of 2 µM forskolin (Sigma-Aldrich). After recording a suitable baseline and, in the case of cells treated with forskolin, luminescence had stabilised (∼30 min), cells were removed from the plate reader to be flashed with light (1 s duration, 470 nm, using a custom-built LED array) at varying intensities (0–15.1 log photon/cm^2^/sec) and were then returned to the reader to record subsequent changes in luminescence (sampled once every 60 s for 30 min).

### BRET assay

HEK293 ΔGs/Gq/G12 KO cells in DMEM were transfected with 500 ng opsin, the appropriate Gα subunit – Gα_s_ (GNAS), 50 ng; Gα_i_(Ser) [a pertussis-toxin-insensitive Gα_i_ (GNAI1) mutant containing the single point mutation Cys351Ser], 200 ng; Gα_q_ (GNAQ), 200 ng – and the following BRET components: 25 ng GRK3–nLuc, 100 ng split-Venus β1 (sVβ1) subunit and 100 ng split-Venus γ2 (sVγ2) (BRET components kindly provided by Professor Kirill Martemyanov, Scripps Research Institute, FL, USA). Cells were incubated overnight with 10 µM 9-*cis* retinal and 100 ng/ml PTX (Sigma-Aldrich). The next day, cells were incubated in L-15 medium (Gibco) for 2 h in the dark at room temperature. Furimazine (Nano-Glo Luciferase Assay Substrate) diluted 1:40 in NanoGlo Luciferase Assay Buffer (both Promega) was added to the cell medium at a 1:5 ratio, and the cells were left for a further 10 min before placing in a plate reader (Optima FLUOStar, BMG) modified to allow ‘in-well’ stimulation with an external light source (CoolLED) via fibre optic. Light emission was recorded at 470 nm and 535 nm (sequential second counts, every 2 s) to allow the BRET ratio to be calculated as raw luminescence at 535 nm/raw luminescence units at 470 nm. After a suitable baseline, cells were stimulated with a 470 nm flash (16.1 log photon/cm^2^/sec, 1 s, CoolLED) from the external light source, and changes in BRET over the subsequent minute were recorded. Data was normalised to the BRET ratio calculated at the timepoint collected immediately prior to light stimulation.

### Statistical analysis

Plate reader recordings (luminescence or BRET ratio) were normalised to the point immediately prior to light stimulus in Microsoft Excel. Statistical analysis, as outlined in the Results and Discussion, was performed in Graphpad Prism. Data were first tested for normality using D'Agostino–Pearson omnibus normality test. Kruskal–Wallis tests with Dunn's multiple comparison post hoc analysis were performed on the average response following light stimulus for data in Fig. 2. ‘*n*’ denotes the number of biological replicates from independent transfections, unless otherwise stated.

### Immunocytochemistry

HEK293T cells were transfected with plasmid expression vectors for hOPN4, musOPN4L or musOPN4S using Lipofectamine 2000 (Thermo Fisher Scientific) and were cultured on poly-L-ornithine-coated glass coverslips before being fixed in 4% paraformaldehyde. Fixed cells were permeabilised in 10% Triton-X100 (Sigma-Aldrich), blocked for 30 min in phosphate-buffered saline (PBS) containing 5% serum (donkey) prior to incubation at room temperature for 1 h with the relevant primary antibody [anti-1D4 mouse IgG (Abcam, AB5417) used at 1:500 for hOPN4, musOpn4S and musOpn4L in Fig. 1A and Fig. S2; anti-mouse Opn4 rabbit polyclonal (ATS-Bio, AB-N39) used at 1:1000 for musOpn4S and musOpn4SL in Fig. 3A] in 1% donkey serum, washed in PBS and incubated for a further 30 min with 10 µg/ml Alexa Fluor 488-conjugated secondary antibody (donkey anti-mouse or anti-rabbit IgG; Life Technologies). Coverslips, mounted in Prolong Antifade Gold medium with DAPI (Invitrogen), were imaged using a Leica DN2500 microscope with DFC365 FX camera (Leica) and a CoolLED pE-400 white light source. Images were acquired using the Leica Application Suite using Chroma A4 (excitation 360 nm, emission 470 nm) and L5 (excitation 480 nm, emission 527 nm) filter sets. Image processing to modulate global contrast and brightness was performed using ImageJ software (NIH, Bethesda, MD, USA).

## Supplementary Material

Supplementary information

Reviewer comments
