# Supplementary material for: Divergent G-protein selectivity across melanopsins from mice and humans
Source: J Cell Sci. 2022 Mar 21;135(6):jcs258474. doi: 10.1242/jcs.258474 (PMC8977054; doi:10.1242/jcs.258474)
Supplement: Supplementary information [file joces-135-258474-s1.pdf]

```
                         1           11          21          31          41          51         60
                         |           |           |           |           |           |          |
sp|Q9UHM6-1|hOPN4        MNPPSGPRVP PSPTQEPSCM ATPAPPSWWD SSQSSISSLG RLPSISPTAP GTWAAAWVPL   60
sp|Q9UHM6-2|hOPN4(v2)    MNPPSGPRVP PSPTQEPSCM ATPAPPSWWD SSQSSISSLG RLPSISPTAP GTWAAAWVPL   60
sp|Q9QXZ9-1|musOpn4L     MDSPSGPRVL SSLTQDPSFT TSPALQGIWN GTQ-NVSVRA QLLSVSPTTS AHQAAAWVPF   59
sp|Q9QXZ9-2|musOpn4S     MDSPSGPRVL SSLTQDPSFT TSPALQGIWN GTQ-NVSVRA QLLSVSPTTS AHQAAAWVPF   59
    h/mOpn4L Chimera     MNPPSGPRVP PSPTQEPSCM ATPAPPSWWD SSQSSISSLG RLPSISPTAP GTWAAAWVPL   60
                         *:.******  .* **:**  ::**  . *: .:* .:*  . :* *:***:. .   ******:

                                           TM1
sp|Q9UHM6-1|hOPN4        PTVDVPDHAH YTLGTVILLV GLTGMLGNLT VIYTFCR--- --------SR SLRTPANMFI  109
sp|Q9UHM6-2|hOPN4(v2)    PTVDVPDHAH YTLGTVILLV GLTGMLGNLT VIYTFCRAVL RGVTVMMQSR SLRTPANMFI  120
sp|Q9QXZ9-1|musOpn4L     PTVDVPDHAH YTLGTVILLV GLTGMLGNLT VIYTFCR--- --------NR GLRTPANMFI  108
sp|Q9QXZ9-2|musOpn4S     PTVDVPDHAH YTLGTVILLV GLTGMLGNLT VIYTFCR--- --------NR GLRTPANMFI  108
    h/mOpn4L Chimera     PTVDVPDHAH YTLGTVILLV GLTGMLGNLT VIYTFCR--- --------SR SLRTPANMFI  109
                         ********** ********** ********** *******            .* .*********

                               TM2                                  TM3
sp|Q9UHM6-1|hOPN4        INLAVSDFLM SFTQAPVFFT SSLYKQWLFG ETGCEFYAFC GALFGISSMI TLTAIALDRY  169
sp|Q9UHM6-2|hOPN4(v2)    INLAVSDFLM SFTQAPVFFT SSLYKQWLFG ETGCEFYAFC GALFGISSMI TLTAIALDRY  180
sp|Q9QXZ9-1|musOpn4L     INLAVSDFLM SVTQAPVFFA SSLYKKWLFG ETGCEFYAFC GAVFGITSMI TLTAIAMDRY  168
sp|Q9QXZ9-2|musOpn4S     INLAVSDFLM SVTQAPVFFA SSLYKKWLFG ETGCEFYAFC GAVFGITSMI TLTAIAMDRY  168
    h/mOpn4L Chimera     INLAVSDFLM SFTQAPVFFT SSLYKQWLFG ETGCEFYAFC GALFGISSMI TLTAIALDRY  169
                         ********** *.*******: *****:**** ********** **:***:*** ******:***

                                              TM4
sp|Q9UHM6-1|hOPN4        LVITRPLATF GVASKRRAAF VLLGVWLYAL AWSLPPFFGW SAYVPEGLLT SCSWDYMSFT  229
sp|Q9UHM6-2|hOPN4(v2)    LVITRPLATF GVASKRRAAF VLLGVWLYAL AWSLPPFFGW SAYVPEGLLT SCSWDYMSFT  240
sp|Q9QXZ9-1|musOpn4L     LVITRPLATI GRGSKRRTAL VLLGVWLYAL AWSLPPFFGW SAYVPEGLLT SCSWDYMTFT  228
sp|Q9QXZ9-2|musOpn4S     LVITRPLATI GRGSKRRTAL VLLGVWLYAL AWSLPPFFGW SAYVPEGLLT SCSWDYMTFT  228
    h/mOpn4L Chimera     LVITRPLATF GVASKRRAAF VLLGVWLYAL AWSLPPFFGW SAYVPEGLLT SCSWDYMSFT  229
                         *********: *  .****:*: ********** ********** ********** *******:**

                                          TM5
sp|Q9UHM6-1|hOPN4        PAVRAYTMLL CCFVFFLPLL IIIYCYIFIF RAIRETGRAL QTFGACKGNG ES-LWQR---  285
sp|Q9UHM6-2|hOPN4(v2)    PAVRAYTMLL CCFVFFLPLL IIIYCYIFIF RAIRETGRAL QTFGACKGNG ES-LWQR---  296
sp|Q9QXZ9-1|musOpn4L     PQVRAYTMLL FCFVFFLPLL IIIFCYIFIF RAIRETGR-- ----ACEGCG ESPLRQRRQW  282
sp|Q9QXZ9-2|musOpn4S     PQVRAYTMLL FCFVFFLPLL IIIFCYIFIF RAIRETGR-- ----ACEGCG ESPLRQRRQW  282
    h/mOpn4L Chimera     PAVRAYTMLL CCFVFFLPLL IIIYCYIFIF RAIRETGRAL QTFGACKGNG ES-LWQR---  285
                         ********    ********* ***:****** ********       **:* * ** * **

                                  TM6                                     TM7
sp|Q9UHM6-1|hOPN4        QRLQSECKMA KIMLLVILLF VLSWAPYSAV ALVAFAGYAH VLTPYMSSVP AVIAKASAIH  345
sp|Q9UHM6-2|hOPN4(v2)    QRLQSECKMA KIMLLVILLF VLSWAPYSAV ALVAFAGYAH VLTPYMSSVP AVIAKASAIH  356
sp|Q9QXZ9-1|musOpn4L     QRLQSEWKMA KVALIVILLF VLSWAPYSTV ALVAFAGYSH ILTPYMSSVP AVIAKASAIH  342
sp|Q9QXZ9-2|musOpn4S     QRLQSEWKMA KVALIVILLF VLSWAPYSTV ALVAFAGYSH ILTPYMSSVP AVIAKASAIH  342
    h/mOpn4L Chimera     QRLQSECKMA KIMLLVILLF VLSWAPYSAV ALVAFAGYAH VLTPYMSSVP AVIAKASAIH  345
                         ****** *** *: *:***** ********:* ********:* :********* **********

                                      H8
sp|Q9UHM6-1|hOPN4        NPIIYAITHP KYRVAIAQHL PCLGVLLGVS RRHSRPYPSY RSTHRSTLTS HTSNLSWISI  405
sp|Q9UHM6-2|hOPN4(v2)    NPIIYAITHP KYRVAIAQHL PCLGVLLGVS RRHSRPYPSY RSTHRSTLTS HTSNLSWISI  416
sp|Q9QXZ9-1|musOpn4L     NPIIYAITHP KYRVAIAQHL PCLGVLLGVS GQRSHPSLSY RSTHRSTLSS QSSDLSWISG  402
sp|Q9QXZ9-2|musOpn4S     NPIIYAITHP KYRVAIAQHL PCLGVLLGVS GQRSHPSLSY RSTHRSTLSS QSSDLSWISG  402
    h/mOpn4L Chimera     NPIIYAITHP KYRVAIAQHL PCLGVLLGVS GQRSHPSLSY RSTHRSTLSS QSSDLSWISG  405
                         ********** ********** **********   ::*:*  ** ********:* ::*:*****

                                               C-TERM
sp|Q9UHM6-1|hOPN4        RRRQESLGSE SEVGWTHMEA AAVWGAAQQA NGRSLYGQGL EDLEAKAPPR PQGHEAETPG  465
sp|Q9UHM6-2|hOPN4(v2)    RRRQESLGSE SEVGWTHMEA AAVWGAAQQA NGRSLYGQGL EDLEAKAPPR PQGHEAETPG  466
sp|Q9QXZ9-1|musOpn4L     RKRQESLGSE SEVGWTDTET TAAWGAAQQA SGQSFCSQNL EDGELKASSS PQVQRSKTPK  462
sp|Q9QXZ9-2|musOpn4S     RKRQESLGSE SEVGWTDTET TAAWGAAQQA SGQSFCSQNL EDGELKASSS PQTK------  456
    h/mOpn4L Chimera     RKRQESLGSE SEVGWTDTET TAAWGAAQQA SGQSFCSQNL EDGELKASSS PQVQRSKTPK  465
                         *:******** ******. *: :*.******* .*:*: .*.* ** * **..  ** :

sp|Q9UHM6-1|hOPN4        ---------- KTKGLIPSQD PR-------- ---------- ---------- ---------M  478
sp|Q9UHM6-2|hOPN4(v2)    ---------- KTKGLIPSQD PR-------- ---------- ---------- ---------M  489
sp|Q9QXZ9-1|musOpn4L     VPGPSTCRPM KGQGARPSSL RGDQKGRLAV CTGLSECPHP HTSQFPLAFL EDDVTLRHLM  522
sp|Q9QXZ9-2|musOpn4S     ---------- ---GHLPSLD LG-------- ---------- ---------- ---------M  466
    h/mOpn4L Chimera     VPGPSTCRPM KGQGARPSSL RGDQKGRLAV CTGLSECPHP HTSQFPLAFL EDDVTLRHLM  525
                                      *   **                                              *
```

Fig. S1. ***Aligned amino acid sequences of hOPN4 and musOpn4 isoforms***
Amino acid sequences of hOPN4 (Q9UHM6-1), hOPN4(v2)(Q9UHM6-2), musOpn4L (Q9QXZ9-1), musOpn4S (Q9QXZ9-2) and h/musOpn4L chimera. Sequences aligned using L-INS-I in MAFFT (v7.475) (Katoh and Standley, 2013). Transmembrane helices indicated in grey. Helix 8 indicated in dark grey. C-terminal tail indicated in blue. "*" indicates positions with fully conserved residue, ":" indicates strongly similar properties between residues, "." Indicates weakly similar properties between residues.

Journal of Cell Science • Supplementary information

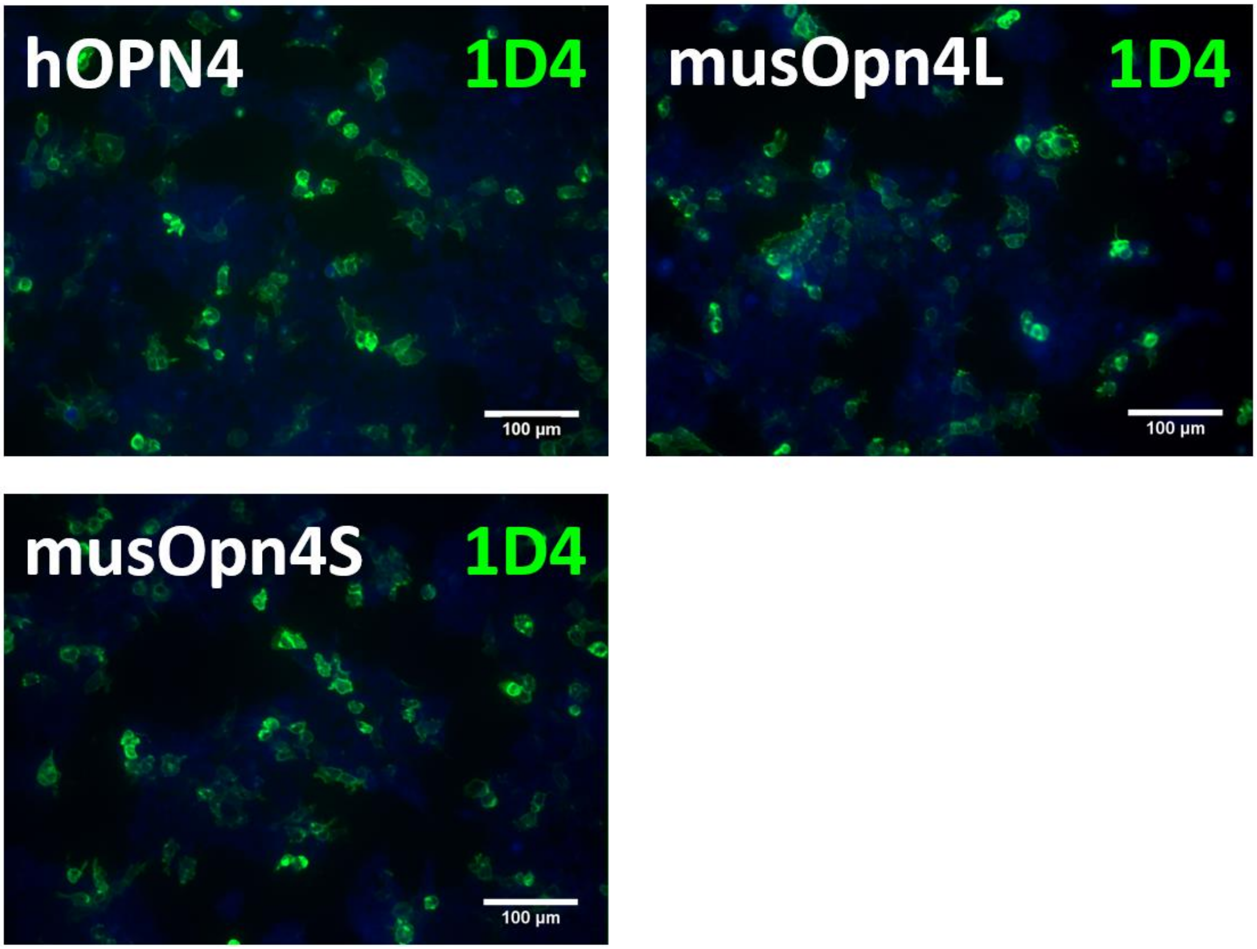

Fig. S2. ***Melanopsin expression in HEK293 cells*** Immunocytochemistry photomicrograph showing expression of hOPN4, musOpn4L, and musOpn4S via staining for 1D4 tag in HEK293 cells (in green). All opsins are expressed well and in cell membranes of HEK293 cells (nuclei stained with DAPI, in blue). Scale bar = 100µM.

Journal of Cell Science • Supplementary information
