# Supplementary material for: Divergent G-protein selectivity across melanopsins from mice and humans
Source: J Cell Sci. 2022 Mar 21;135(6):jcs258474. doi: 10.1242/jcs.258474 (PMC8977054; doi:10.1242/jcs.258474)
Supplement: Reviewer comments [file joces-135-258474_review_history.pdf]

Journal of Cell Science

Divergent G-protein selectivity across melanopsins from mice and humans

Richard J. McDowell, Jessica Rodgers, Nina Milosavljevic and Robert J. Lucas
DOI: 10.1242/jcs.258474
Editor: John Heath

Review timeline

Original submission:	28 January 2021
Editorial decision:	14 April 2021
First revision received:	7 January 2022
Accepted:	7 February 2022

Original submission

First decision letter

MS ID#: JOCES/2021/258474

MS TITLE: Divergent G-protein selectivity across melanopsins from mice and humans.

AUTHORS: Richard J McDowell, Nina Milosavljevic, and Robert Lucas
ARTICLE TYPE: Short Report

We have now reached a decision on the above manuscript.

To see the reviewers' reports and a copy of this decision letter, please go to: https://submit-jcs.biologists.org and click on the 'Manuscripts with Decisions' queue in the Author Area. (Corresponding author only has access to reviews.)

As you will see, the reviewers raise a number of criticisms that prevent me from accepting the paper at this stage. They suggest, however, that a revised version might prove acceptable, if you can address their concerns. If you think that you can deal satisfactorily with the criticisms on revision, I would be pleased to see a revised manuscript. We would then return it to the reviewers.

We are aware that you may be experiencing disruption to the normal running of your lab that makes experimental revisions challenging. If it would be helpful, we encourage you to contact us to discuss your revision in greater detail. Please send us a point-by-point response indicating where you are able to address concerns raised (either experimentally or by changes to the text) and where you will not be able to do so within the normal timeframe of a revision. We will then provide further guidance. Please also note that we are happy to extend revision timeframes as necessary.

Please ensure that you clearly highlight all changes made in the revised manuscript. Please avoid using 'Tracked changes' in Word files as these are lost in PDF conversion.

I should be grateful if you would also provide a point-by-point response detailing how you have dealt with the points raised by the reviewers in the 'Response to Reviewers' box. Please attend to all of the reviewers' comments. If you do not agree with any of their criticisms or suggestions please explain clearly why this is so.

© 2022. Published by The Company of Biologists under the terms of the Creative Commons Attribution License (https://creativecommons.org/licenses/by/4.0/).

<u>Reviewer 1</u>

Advance summary and potential significance to field

advance: clarification of the G protein coupling specificities of three opto GPCRs and identification of the particular variant that is most ideal for optogenetic Gq activation. This is a major advance for the application of melanopsins to stimulate specific signaling pathways.

Comments for the author

very well written paper, very good to read, logical, interesting, and most conclusions entirely supported by data. In a few instances the paper would benefit from additional clarification and some small extra experiments that would clearly enhance the impact of this work further.

Specific comments:
- Materials&Methods, line 234, please correct „reporters instead of reproters“
- Lines 249-252: please write down how long cells need for luminescence stabilisation after adding forskolin, this is a detail of interest for replicating your data.
- Figure 1 : Bii is missing, please correct
- lines 78/79: it would be helpful to mention that cells responded with a reduction of luminescence after forskolin stimulation. Please add these few words to enhance clarity of the data.
- lines 84-87: would it be helpful for the readers to mention and comment on the kinetics of the overshoot glosensor luminescence?
- line 93: HEK293s what does the „s“ stand for?
- Figure 2B: what happens in cells expressing „no opsin“? Why is there no flat line? Does the light flash by itself trigger alpha-betagamma dissociation and if so, why is this happening? Please comment on this briefly in the text.
- lines 126/127: is there any explanation for the small amplitude achieved with OPN4 in Gai(ser) expressing cells? Which Gai is used here? PLease indicate the isoform. Would Gao be the better BRET partner to show bg-GRK interaction? Whether OPN4 prefers Go over Gi is not evident from Fig1 but it is an obvious question resulting from the unexpectedly small amplitudes in Fig2D for OPN4. It could be interesting extra information revealing intra-Gi/o family preferences for the different opsins.
- lines 137/138. You state „Both isoforms of mouse melanopsin express well in HEK293 cells (Figure 3A).“ Please state whether expression levels are comparable expressing well is a bit vague. Since coupling specificity is directly related to overall expression, I would explicitly mention what is important here. I would also recommend to show a zoom out view (not only one cell) to allow judgement of expression quantity (could be added as suppl figure).
- lines 138/139. You state „Interestingly, though, neither closely replicated the biphasic GloSensor flash response produced by hOPN4 in these cells (Figure 3B).“
Is this really true? PLease look at the y-axis scaling, how does the potential Gi response look like at lower light intensities once you adjust the scale? The downward-deflected signals drown in the positive response, but is there really nothing or only nothing because of axis scaling? A zoom-in with adjusted axis will help to resolve this.
-musOPN4S: from the one cell image in Fig3A 4L and 4S appear similar but the data in Bii imply either very poor functionality or very poor expression. Are the effects correlated with the light intensities? The scatter is huge interpretation very hard. Would repetitions increase confidence in the data? Is there any indication for bellshaped effects or are the small decreases in luminescence more random than light-intensity-related?
-please explain why you see decrease in luminescence in Fig3Bii for musOPN4S but no indication for Gi activity in Dii?
- can we exclude that OPN4S is a receptor that is meant by nature to be poorly functional to act as dominant negative inhibitor for OPN4L? Or is it poorly functional because its expression is too low to resolve function properly? This also relates to my above comment on the images showing expression of one or two cells only. It would help to figure out the purpose of this protein, is its presence good for signaling or for the inhibition of signaling of 4L?
- in direct relation to the above comment this reviewer realizes that Fig4D is used to argue that differences in expression likely do not account for different Gs signaling functionality because EC50 values are comparable. I agree but it is hard to see curve fits for musOPN4S in Fig4Aiv (it looks dead the way it is presented) and makes solid expression data even more important (used by the

© 2022. Published by The Company of Biologists under the terms of the Creative Commons Attribution License (https://creativecommons.org/licenses/by/4.0/).

authors as argument that Gs engagement differs but not expression). Please improve the data to make a solid expression claim (for example by showing zoom out images of Fig3A and quantification of such). And, please consider whether a split y-axis would be helpful to show that musOPN4S is really giving a functional response with comparable potency to that of the other 2 opsins. The table includes the info but the green triangles do not support functionality optically.
- In relation to Fig4 the authors suggest that the extended C-terminal tail likely explains why musOPN4L loves to interact with Gs, distinct from musOPN4S and hOPN4. If the tail is the molecular determinant of Gs signaling, then a tail swap to OPN4S at a minimum and/or OPN4 would help to clarify two main questions:
(i) whether Gs activity is really encoded in the tail, and (ii) whether poor Gs signaling is really unrelated to expression but to poor function (of mus OPN4S).
A tail swap could be a minimal but extra experiment to adress this very helpful issue. Conversely, tail swap of the 4L tail to the 4S receptor should decrease Gs activity only but retain Gq and Gi signaling.

Reviewer 2

Advance summary and potential significance to field

The paper by McDowell, Milosavljevic and Lucas has made significant advances to our understanding of mammalian melanopsin and the phototransduction cascade in intrinsic photosensitive retinal ganglion cells (iPRGCs). Melanopsin is a R-type visual pigment and the conventional wisdom until recently was that the biochemical cascade activated by melanopsin was a Gq mediated cascade similar to the one found in drosophila photoreceptors. It was also known that melanopsin could activate Gi G-proteins even though it was thought they were not the opsin's cognate G- protein. This demonstrating that melanopsin was a "promiscuous" GPCR. Recent papers have suggested that our conventional understanding of melanopsin's phototransduction cascade is not the complete story. Jiang et al 2018 suggests that two sub types of iPRGCs use a cyclic nucleotide gated channel in their photo transduction cascade. This paper demonstrated using tissue culture cells that melanopsin can activate Gs as well as Gq/11 and Gio . This is important as it provides a mechanism to regulate cyclic nucleotide gated channels. The other advance made in this paper is to demonstrate that the two isoforms of mouse melanopsin have different affinities for G-Proteins. These 2 isoforms are differentially expressed in iPRGC subtypes, thus providing a mechanism for differential phototransduction cascade.

Comments for the author

I recommend that the paper McDowell, Milosavljevic and Lucas be published with the following minor revisions.
1. The authors use HEK delta Gs/Gq/G12 knockout cells. They do not describe the origin of these cells.
2. The use 100 ng of pertussis toxin to inhibit Gi. Please provide a reference or evidence that this concentration is sufficient to ribosylate all the Gi.
3. The authors should refer the reader to their intensity response curves in Fig.4 for this reviewer wanted to see the data of Bi and Bii displayed in such a fashion. It was only in the last figure did I find this graph.
4. In the figure legend of Fig. 1 please indicate that the light flash used was 470 nM.
5. In figure 2 can the authors explain the light dependent increase in BRET under conditions where there was no opsin transfected.
6. In Figure 3 please indicate that the error bars are S.E.M. (I presume).
The authors should address that fact that the data using the short isoform is very variable. Are the data om Bii significant

© 2022. Published by The Company of Biologists under the terms of the Creative Commons Attribution License (https://creativecommons.org/licenses/by/4.0/).

First revision

Author response to reviewers' comments

We are grateful for the referees' valuable comments on our manuscript. We apologise for the delay in returning our revised manuscript, which in part reflects disruptions caused by the Covid pandemic and part the substantial additional data we have collected in response to the reviewers' comments.

Reviewer 1 Comments for the Author:

Specific comments:

Materials&Methods, line 234, please correct „reporters instead of reproters" – **Done**

Lines 249-252: please write down how long cells need for luminescence stabilisation after adding forskolin, this is a detail of interest for replicating your data. – **Text has now been updated to include (in yellow): Line 282-284** – "They were then incubated with 2mM beetle luciferin (Sigma-Aldrich) substrate at room temperature for 30 minutes before being transferred to the plate reader"

Figure 1 : Bii is missing, please correct **- The Figure has now been updated to remove Bii, and instead list hRod Opsin data under C.**

lines 78/79: it would be helpful to mention that cells responded with a reduction of luminescence after forskolin stimulation. Please add these few words to enhance clarity of the data. – **The text has now been updated on line 80 to include (new text in yellow):** "HEK293T cells expressing GloSensor and human melanopsin (hOPN4; Figure 1A) responded to a 1s 470nm light flash (at intensities of ≤12.08 log photon/cm^2/sec) with a reduction in luminescence following pre-treatment with forskolin (Figure 1 B), as previously reported…"

lines 84-87: would it be helpful for the readers to mention and comment on the kinetics of the overshoot glosensor luminescence? – **A brief comment has been added on the kinetics of overshoot on line 84.**

line 102: HEK293s what does the „s" stand for? – **The text has been updated to "HEK293 cells"**

Figure 2B: what happens in cells expressing „no opsin"? Why is there no flat line? Does the light flash by itself trigger alpha-betagamma dissociation and if so, why is this happening? Please comment on this briefly in the text. – **We do not have a full explanation for this phenomenon, it could reflect an intrinsic light response of HEK293 cells, but our best guess is that it reflects some bleaching of the BRET components by our stimulating light. We know that both NanoLuc and Venus will be sensitive to light. We do not have a validated explanation for why this should result in a change in BRET ratio, but the magnitude of the change is small and it is hard to exclude the possibility that it reflects alterations in activity of the two BRET components.**
We have therefore changed the text to highlight this potential explanation (new text in yellow):
Line 120: We found that light induced a small change in BRET even in cells lacking opsin (possibly reflecting partial bleaching of components of the BRET assay, or some intrinsic light response of HEK293 cells). Nevertheless, a light flash reliably induced a much larger increase in BRET in hOPN4 expressing cells,

lines 126/127: is there any explanation for the small amplitude achieved with OPN4 in Gai(ser) expressing cells? Which Gai is used here? PLease indicate the isoform. Would Gao be the better BRET partner to show bg-GRK interaction? Whether OPN4 prefers Go over Gi is not evident from Fig1 but it is an obvious question resulting from the unexpectedly

© 2022. Published by The Company of Biologists under the terms of the Creative Commons Attribution License (https://creativecommons.org/licenses/by/4.0/).

small amplitudes in Fig2D for OPN4. It could be interesting extra information revealing intra-Gi/o family preferences for the different opsins. – **The small amplitude of BRET ratio change reflects a relatively weaker interaction of hOPN4 with Gai than is observed for Rod Opsin. Rod Opsin signals very strongly through Gi and so a lower amplitude response does not necessarily indicate that hOPN4 signals weakly through Gi, but rather that is just weaker than Rod Opsin in this instance. The specific subunit used in this data is GNAI-1. The methods have been updated to include this information (Line 295). It is possible that Gao could provide a better response for hOPN4. To conduct a full investigation of all subunits capable of targeting the Gio pathway would require the additional production of pertussis toxin insensitive mutants for Gi2, Gi3, GoA and GoB. While interesting, we feel this additional work is beyond the scope of the paper.**

lines 152/153. You state „Both isoforms of mouse melanopsin express well in HEK293 cells (Figure 3A).“ Please state whether expression levels are comparable, expressing well is a bit vague. Since coupling specificity is directly related to overall expression, I would explicitly mention what is important here. I would also recommend to show a zoom out view (not only one cell) to allow judgement of expression quantity (could be added as suppl figure). **We have been happy to provide zoomed out images as requested, which are included in Supplementary Figure 2. We have also undertaken an explicit exploration of the impact of opsin expression level on experimental outcome in response to this reviewer's further queries (see below).**

lines 153/154. You state „Interestingly, though, neither closely replicated the biphasic GloSensor flash response produced by hOPN4 in these cells (Figure 3B).“ Is this really true? Please look at the y-axis scaling, how does the potential Gi response look like at lower light intensities once you adjust the scale? The downward-deflected signals drown in the positive response, but is there really nothing or only nothing because of axis scaling? A zoom-in with adjusted axis will help to resolve this. - **We thank the referee for highlighting this area of potential confusion. On their recommendation we now include a zoom in, which shows the small decrease in cAMP at lowe light intensities. We have also changed the text to '…neither replicated the <u>marked</u> biphasic …'The next sentence in the manuscript then specifically addresses the cAMP reduction at low intensities (Lines 148-151).**

musOPN4S: from the one cell image in Fig3A 4L and 4S appear similar but the data in Bii imply either very poor functionality or very poor expression. Are the effects correlated with the light intensities? The scatter is huge, interpretation very hard. Would repetitions increase confidence in the data? Is there any indication for bellshaped effects or are the small decreases in luminescence more random than light-intensity-related?

please explain why you see decrease in luminescence in Fig3Bii for musOPN4S but no indication for Gi activity in Dii can we exclude that OPN4S is a receptor that is meant by nature to be poorly functional to act as dominant negative inhibitor for OPN4L? Or is it poorly functional because its expression is too low to resolve function properly? This also relates to my above comment on the images showing expression of one or two cells only. It would help to figure out the purpose of this protein, is its presence good for signaling or for the inhibition of signaling of 4L? -

in direct relation to the above comment this reviewer realizes that Fig4D is used to argue that differences in expression likely do not account for different Gs signaling functionality because EC50 values are comparable. **I agree** but it is hard to see curve fits for musOPN4S in Fig4Aiv (it looks dead the way it is presented) and makes solid expression data even more important (used by the authors as argument that Gs engagement differs but not expression). Please improve the data to make a solid expression claim (for example by showing zoom out images of Fig3A and quantification of such). And, please consider whether a split y-axis would be helpful to show that musOPN4S is really giving a functional response with comparable potency to that of the other 2 opsins. The table includes the info but the green triangles do not support functionality optically. -

© 2022. Published by The Company of Biologists under the terms of the Creative Commons Attribution License (https://creativecommons.org/licenses/by/4.0/).

The referee's point about the potential impact of expression level on our findings was well taken, and we decided to explicitly address this potential confound. While all of our opsins express well at the level of immunocytochemistry (As now demonstrated in Supplementary Figure 2), it is fundamentally impossible to use this (or any protein quantification methodology) to predict the amount of *functional* opsin. Protein may be produced and even locate to the plasma membrane but not take up chromophore or be signalling active, as both of those characteristics require appropriate folding and processing.
Rather than put more effort into quantifying opsin expression levels, we therefore explored the effect of changing the concentration of opsin expression plasmid used for transfection. We reasoned that alterations in this parameter would de facto change the amount of opsin expressed and allow us to determine whether our conclusions are robust to alterations in opsin expression. This data is now presented in Fig 4A and described in the appropriate point in the results section (Lines 151-184).
We did indeed find that the amplitude of light responses was impacted by plasmid concentration, confirming that this manipulation had effectively altered the amount of opsin across a physiologically relevant range. When corrected in this way, the difference in signalling preference between mouse Opn4L and Opn4S was lost, indicating that this had indeed been produced by differences in expression efficiency. Conversely, however, the large difference in Gio vs Gs activity in hOPN4 vs the 2 mouse melanopsins was retained. We have adjusted the presentation of our data and the conclusions drawn accordingly and thank the referee for highlighting what turned out to be such an important omission from our initial submission.

In relation to Fig4 the authors suggest that the extended C-terminal tail likely explains why musOPN4L loves to interact with Gs, distinct from musOPN4S and hOPN4. If the tail is the molecular determinant of Gs signaling, then a tail swap to OPN4S at a minimum and/or OPN4 would help to clarify two main questions:
(i) whether Gs activity is really encoded in the tail, and (ii) whether poor Gs signaling is really unrelated to expression but to poor function (of mus OPN4S). A tail swap could be a minimal but extra experiment to address this very helpful issue. Conversely, tail swap of the 4L tail to the 4S receptor should decrease Gs activity only but retain Gq and Gi signaling. -
Given the new data showing that the signalling capacity of mOpn4l and s are similar, the suggested tail swaps between the mouse isoforms are no longer necessary. A related question is whether there might be isoforms of hOPN4 with 'mouse-like' Gs signalling. We have now addressed this issue in the context of two potential structural isoforms of human melanopsin. The first is one proposed by computational analysis of likely splicing events (differing in composition of IL1; termed hOPN4v2). For the second we were interested in whether a long isoform of hOPN4 (if it existed) may have different signalling. To test this we added the C-terminal tail extension of mOpn4L (which we know to be compatible with a functional protein) to hOPN4 to generate a h/mOPN4L chimera. We failed to record light responses using hOPN4v2, suggesting it may be non-functional, while signalling from h/mOPN4L was similar to that of hOPN4, consistent with the view that the C-terminus extension does not alter g-protein selectivity. We believe that these experiments add confidence that melanopsin in humans has lower Gs signalling capacity.

Reviewer 2 Comments for the Author:

1. The authors use HEK delta Gs/Gq/G12 knockout cells. They do not describe the origin of these cells. **- We apologise for this important omission, the manuscript text has now been updated to include the source of these cells on line 278: "Kindly provided by Prof. Asuka Inoue, Tohoku University"**

2. The use 100 ng of pertussis toxin to inhibit Gi. Please provide a reference or evidence that this concentration is sufficient to ribosylate all the Gi. – **This concentration of**

© 2022. Published by The Company of Biologists under the terms of the Creative Commons Attribution License (https://creativecommons.org/licenses/by/4.0/).

Pertussis toxin is sufficient to irradicate the cAMP decrease observed in Gs/Gq/G12 Knockout HEK293 cells (as observed in Figure 1H vs 1F cells lacking exogenous Gs, and 3Cii & Dii vs 3Ciii & Diii cells lacking exogenous Gs). Given that this cAMP reduction is driven solely by Gio in this knockout line, it therefore follows that the concentration of PTX is capable of ribosylating the majority of Gai and Gao subunits to the extent that Gio signalling is abolished. This concentration of pertussis toxin has been previously published and we have made note of this in the methods on Lines 284-285 "…and, where relevant, 100ng/ml Pertussis toxin (PTX) (Sigma-Aldrich) (as described previously in Bailes & Lucas 2013)."

3. The authors should refer the reader to their intensity response curves in Fig.4 for this reviewer wanted to see the data of Bi and Bii displayed in such a fashion. It was only in the last figure did I find this graph.
   The irradiance response data has been moved to Figure 1 (and 3) allowing us to refer to them at the appropriate points in the text.

4. In the figure legend of Fig. 1 please indicate that the light flash used was 470 nM. – **The figure legend has now been updated with this information.**

5. In figure 2 can the authors explain the light dependent increase in BRET under conditions where there was no opsin transfected. **- We do not have a validated explanation, but a parsimonious explanation (which is hard to exclude) is that the small increase in BRET reflects bleaching of the BRET components. We have modified the text accordingly (please see response to similar point raised by reviewer 1).**

6. In Figure 3 please indicate that the error bars are S.E.M. (I presume). – **This has now been added to the figure legend.**

7. The authors should address that fact that the data using the short isoform is very variable. Are the data in Bii significant?
   As highlighted in our response to reviewer 1 we have now collected a new dataset comparing the mouse isoforms that addresses the issues highlighted with response variability of the short murine isoform.

<u>Second decision letter</u>

MS ID#: JOCES/2021/258474

MS TITLE: Divergent G-protein selectivity across melanopsins from mice and humans.

AUTHORS: Richard J McDowell, Jessica Rodgers, Nina Milosavljevic, and Robert Lucas
ARTICLE TYPE: Short Report

I am happy to tell you that your manuscript has been accepted for publication in Journal of Cell Science, pending standard ethics checks.

<u>Reviewer 1</u>

Advance summary and potential significance to field

please see below, everything is said in the comments to the authors

Comments for the author

I have already commented on the value of this manuscript in my earlier report. I highly appreciate the efforts of the authors and their explanations and have no objection against publishing their

© 2022. Published by The Company of Biologists under the terms of the Creative Commons Attribution License (https://creativecommons.org/licenses/by/4.0/).

paper in its present form. With the new experimentation and interpretation this is a valuable addition to the scientific literature. I thank the authors for their effort and for presenting science so well that it's fun to read.

Reviewer 2

Advance summary and potential significance to field

The paper by McDowell, Milosavljevic and Lucas has made significant advances to our understanding of mammalian melanopsin and the phototransduction cascade in intrinsic photosensitive retinal ganglion cells (iPRGCs). Melanopsin is a R-type visual pigment and the conventional wisdom until recently was that the biochemical cascade activated by melanopsin was a Gq mediated cascade similar to the one found in drosophila photoreceptors. It was also known that melanopsin could activate Gi G-proteins even though it was thought they were not the opsin's cognate Gprotein.
This result demonstrated that melanopsin was a "promiscuous" GPCR. Recent papers have suggested that our conventional understanding of melanopsin's phototransduction cascade is not the complete story. Jiang et al 2018 suggests that two sub types of iPRGCs use a cyclic nucleotide gated channel in their photo transduction cascade. This paper demonstrated using tissue culture cells that melanopsin can activate Gs as well as Gq/11 and Gio . This is important as it provides a mechanism to regulate cyclic nucleotide gated channels. An other advance made in this paper is to demonstrate that the two isoforms of mouse melanopsin have different affinities for G-Proteins. These 2 isoforms are differentially expressed in iPRGC subtypes, thus providing a mechanism for differential phototransduction cascade. Finally, the revised manuscript details a difference in G-protein activation of human and mouse melanopsin. This is a fascinating result, suggesting that one has to careful using the mouse as a model for primate and human activity.

Comments for the author

The revised manuscript should be published after 2 minor corrects:
1. In figure 4 the authors refer to ng of opsin. This is incorrect, rather the figure should read ng of opsin cDNA.
2. Could the authors please clarify what they mean by a "technical replicate".

© 2022. Published by The Company of Biologists under the terms of the Creative Commons Attribution License (https://creativecommons.org/licenses/by/4.0/).
